# Electrographic Changes Accompanying Recurrent Seizures under Ketogenic Diet Treatment

**DOI:** 10.3390/ph10040082

**Published:** 2017-10-20

**Authors:** Chiara Lucchi, Maddalena Marchiò, Elisa Caramaschi, Carmela Giordano, Rocco Giordano, Azzurra Guerra, Giuseppe Biagini

**Affiliations:** 1 Laboratory of Experimental Epileptology, Department of Biomedical, Metabolic and Neural Sciences, University of Modena and Reggio Emilia, 41125 Modena, Italy; chiara.lucchi@unimore.it (C.L.); maddalena.marchio@unimore.it (M.M.); carmela.giordano@unimore.it (C.G.); roccogio93@gmail.com (R.G.); 2 Pediatric Neurology, Polyclinic Hospital, 41124 Modena, Italy; caramaschi.elisa@policlinico.mo.it (E.C.); azzurra.guerra@unimore.it (A.G.); 3 Azienda Unità Sanitaria Locale di Modena, 41121 Modena, Italy; 4 Center for Neuroscience and Neurotechnology, University of Modena and Reggio Emilia, 41125 Modena, Italy

**Keywords:** 6-Hz corneal stimulation, electroencephalography, ketogenic diet, Lennox-Gastaut syndrome, lissencephaly, seizure

## Abstract

The ketogenic diet (KD) is increasingly used to treat epilepsy refractory to antiepileptic drugs and other neurological disorders. In animal models, the KD was found to increase the threshold to seizures induced by different convulsive stimulations. However, in models in which suprathreshold stimuli were used, a paradoxical seizure worsening was consistently observed in KD-fed animals. To better define this phenomenon, we characterized the electrographic response to seizures induced in mice which were treated with the KD, and then corneally stimulated at 6-Hz in four different sessions. We also evaluated the electroencephalogram (EEG) in three patients in which the KD was associated with a paradoxical worsening of epileptic seizures. Although seizures were initially less severe, a remarkable prolongation of the electrographic response was observed in mice receiving the KD from the second session of 6-Hz corneal stimulation and onwards. The EEG was also markedly altered in the presence of progressive seizure aggravation observed in children treated with the KD, specifically one affected by Lennox–Gastaut syndrome and two by type I lissencephaly. These results suggest that when seizures are induced or recur because of resistance to therapeutic interventions, the KD may change the EEG by potentiating the electrographic epileptic activity.

## 1. Introduction

Epilepsy affects approximately 70 million patients worldwide [1]. Although a variety of antiepileptic drugs (AEDs) are currently available to address this neurological disorder, up to one-third of people affected by epilepsy do not properly respond to AEDs and present increased risk of premature death [2]. This major neurological problem may be partly addressed by treating patients who are refractory to AEDs with a ketogenic diet (KD) [3]. This nutritional approach has been proved to be effective in most drug-resistant pediatric patients [4] and, according to a recent meta-analysis [5], reduction in seizure frequency is observed in the vast majority of children maintained on a typical KD for at least three months. Additionally, the KD is used as a first line intervention in patients affected by GLUT-1 deficiency syndrome and pyruvate dehydrogenase complex deficiency (PDCD), who experience difficult-to-treat seizures because of reduced glucose transport or metabolism [5]. Moreover, ketone bodies have been considered as a tool to address the reduced glucose uptake occurring in the brain of patients affected by Alzheimer’s disease [6].

In spite of the recognized efficacy of the KD, the clinical use of this nutritional approach is not so widespread as could be expected [7]. One of the reasons for this limited use could be found in the considerable high dropout rates reported in patients maintained on the KD [8]. In addition, adverse events may also be encountered during the initiation period of the KD, the most frequent of which are represented by gastrointestinal disorders, reported to occur in one-third or more of the treated children [9]. Occasionally, kidney stones and metabolic disorders may also occur during KD administration. A further reason for dropping out of the KD could be the lack of positive response to the treatment, a phenomenon which is especially encountered in the long-term [8]. On the other hand, dropouts due to increased seizure frequency or severity have not been reported in patients maintained on the KD [8], although paradoxical seizure worsening is instead observed in patients affected by epilepsy and treated with AEDs [10].

A different scenario emerged from studies based on animal models. Although the KD has been consistently shown to increase the threshold of seizure induction, lack of effects or paradoxical seizure worsening were instead reported when suprathreshold stimuli were applied [11,12,13,14]. The variability found in animal models has tentatively been explained by considering the specific protocol used to induce the seizures when testing the KD effects [14]. For instance, seizures were more severe in rodents maintained on the KD and exposed to kainate-induced *status epilepticus* or to a maximal electroshock [14,15], whereas the KD was consistently found to be protective in the 6-Hz corneal stimulation test [16,17]. As this latter test is thought to model seizures refractory to AEDs [18,19,20], it appears particularly interesting that the KD may increase the threshold to 6-Hz corneally induced seizures.

In view of the positive results obtained by investigating the KD in two different kindling models, as well as in the pilocarpine model [21,22], a further possible therapeutic application of this nutritional treatment has been suggested for epileptogenesis. However, when performing similar experiments, other investigators observed partial effects [23] or no results [24]. As we recently proposed a model of repeated 6-Hz corneal stimulation in which seizures were found to progressively increase in severity, so to prefigure an epileptogenic process [25,26,27], we aimed at evaluating the KD effects in this model. Specifically, we decided to carefully monitor the KD effects on the progression in seizure severity caused by repeating the seizure induction, as well as to assess the KD effects on electrographic discharges recorded from mice exposed to the four different sessions of 6-Hz corneal stimulation. Additionally, we retrospectively examined patients maintained on the KD to evaluate whether the observations made in the animal model could be clinically relevant.

## 2. Results

### 2.1. Seizure Worsening after an Initial Beneficial Effect of KD Administration in Mice

**Figure 1:**
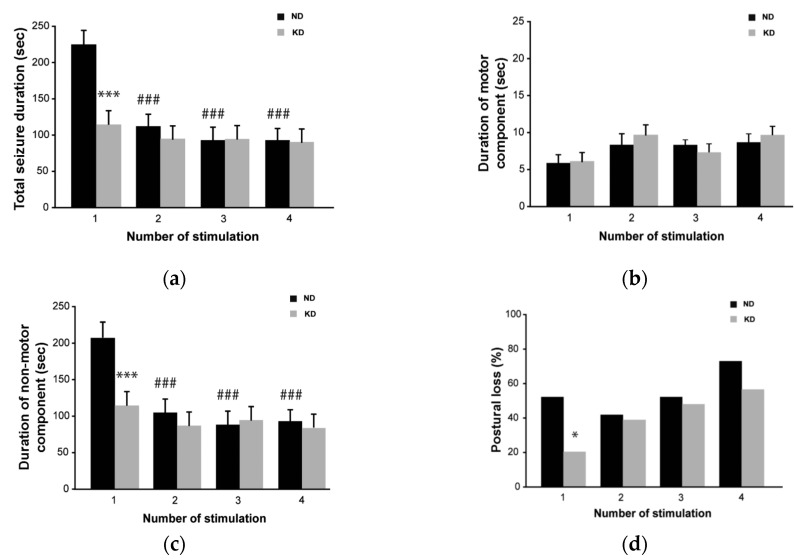
Behavioral changes during repeated 6-Hz corneal stimulation in mice fed the ketogenic diet (KD). (**a**) Seizure duration, considered as the overall period including both motor (convulsions) and non-motor (stunned posture) components of the seizure, was significantly shorter in KD vs. control mice at the first session (*** *p* < 0.001; Holm–Šídák test); however, after the first session the seizure duration decreased in control mice (### *p* < 0.001 for sessions 2, 3 and 4 vs. session 1) and no differences were observed between the two groups. (**b**) Histograms illustrating the mean duration of the motor component of the seizure. Note that no differences were found between the two treatment groups. (**c**) Histograms illustrating the mean duration of the non-motor component of the seizure, which was significantly shorter in KD vs. control mice only at the first session of corneal stimulation (*** *p* < 0.001). Note that this component decreased in control mice from the second to the last session (### *p* < 0.001 vs. session 1). For this reason, no treatment-related differences were observed in sessions 2–4. (**d**) Note that the KD significantly reduced the number of animals developing generalized tonic-clonic convulsions with postural loss at the first seizure induction (* *p* < 0.05, Fisher’s exact test). However, this difference was not appreciated in further sessions; *n* = 9 for the normal diet (ND) group; *n* = 9 for KD group. Statistical significance values are indicated as *: *p* < 0.05, ***: *p* < 0.001, ###: *p* < 0.001.

Mice maintained on the KD developed a significant ketosis, as β-hydroxybutyric acid levels increased three-fold (1.7 ± 0.1 mmol/mL, mean ± standard error of the mean (SEM); *p* < 0.01, Student’s *t* test) with respect to controls (0.5 ± 0.05 mmol/mL). By visual inspection, the first seizure induced by 6-Hz stimulation was shorter in KD-treated mice compared to control mice (114.7 ± 15.1 vs. 211.2 ± 31.7 s; *p* < 0.001, Holm–Šídák test; Figure 1a). However, no differences in duration were found between the two groups in the following session because seizures shortened significantly in controls (113.9 ± 16.4 s in session 2, *p* < 0.001 vs. session 1 of control group), as expected [25,26,27]. Consistently, the change in seizure duration was caused by the shortening of the non-motor component of the seizure, corresponding to stunned behavior, whereas the motor component did not change in both groups (Figure 1b,c). More importantly, the seizure severity, evaluated as the percentage of animals presenting generalized tonic-clonic convulsions with postural loss, was significantly higher in control mice (56% compared to 20% in mice fed the KD; *p* < 0.05, Fisher’s exact test) during the first seizure (Figure 1d). Again, no differences were found in the other sessions as an increasing number of mice under KD developed generalized tonic-clonic convulsions. None of the animals developed spontaneous seizures and no seizure was observed in the interstimulation period or even after the four sessions of seizure induction.

**Figure 2:**
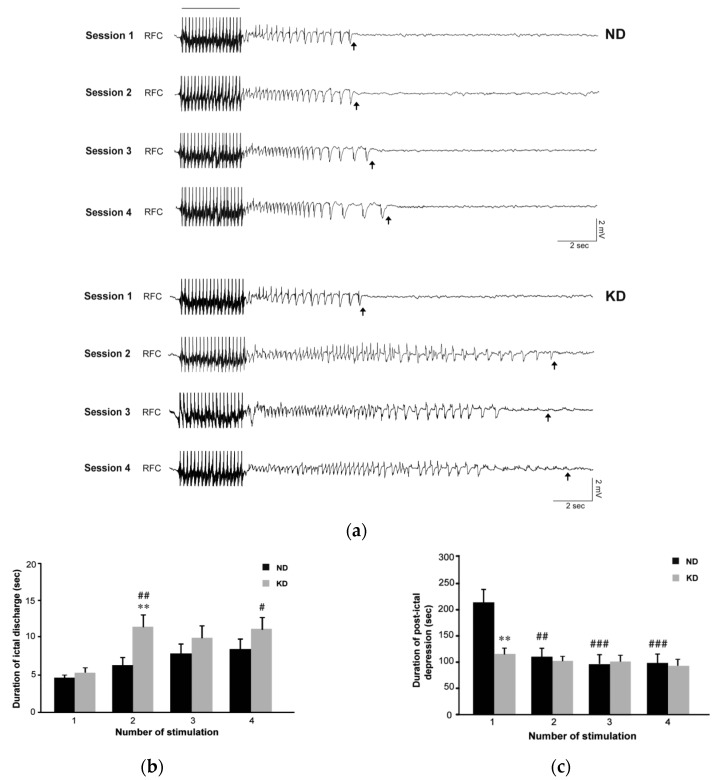
Video electrocorticographic (ECoG) recordings in mice fed the ketogenic diet (KD) and repeatedly tested in the 6-Hz corneal stimulation model. (**a**) ECoG traces illustrating the response detected in the presence of the normal diet (ND) or the KD, during and after a corneal stimulation of one representative mouse after repeated electrostimulations. The black bar indicates a stimulus artifact. Black arrows indicate the end of the ictal discharge. Note that the epileptiform activity changed in terms of duration and waveform when comparing session 1 with session 2. (**b**) Histograms illustrating the mean duration of ictal discharges recorded in session 1 and 2 in ND (*n* = 9) and KD (*n* = 9) groups. The duration of ictal discharges significantly increased in KD mice at the second session of stimulation (## *p* < 0.01 for session 2 vs. session 1; Holm–Šídák test). Moreover, these values were significantly higher than in ND mice when considering session 2 (** *p* < 0.01). Interestingly, a longer ictal activity was observed in the KD group in the subsequent sessions of seizure induction. In particular, a statistical difference was found by comparing session 4 to session 1 in the same animals (# *p* < 0.05; Holm–Šídák test). (**c**) Histograms illustrating the mean duration of post-ictal depression as measured in the various sessions of 6-Hz corneal stimulation. The duration of post-ictal depression was significantly reduced in the ND group at the second session of stimulation (## *p* < 0.01, sessions 2 vs. session 1). Note also that at the first session, post-ictal depression was shorter in KD mice compared to ND mice (** *p* < 0.01). RFC denotes right frontal cortex (epidural electrode). Statistical significance values are indicated as #: *p* < 0.05, **: *p* < 0.01, ##: *p* < 0.01, ###: *p* < 0.001.

**Figure 3:**
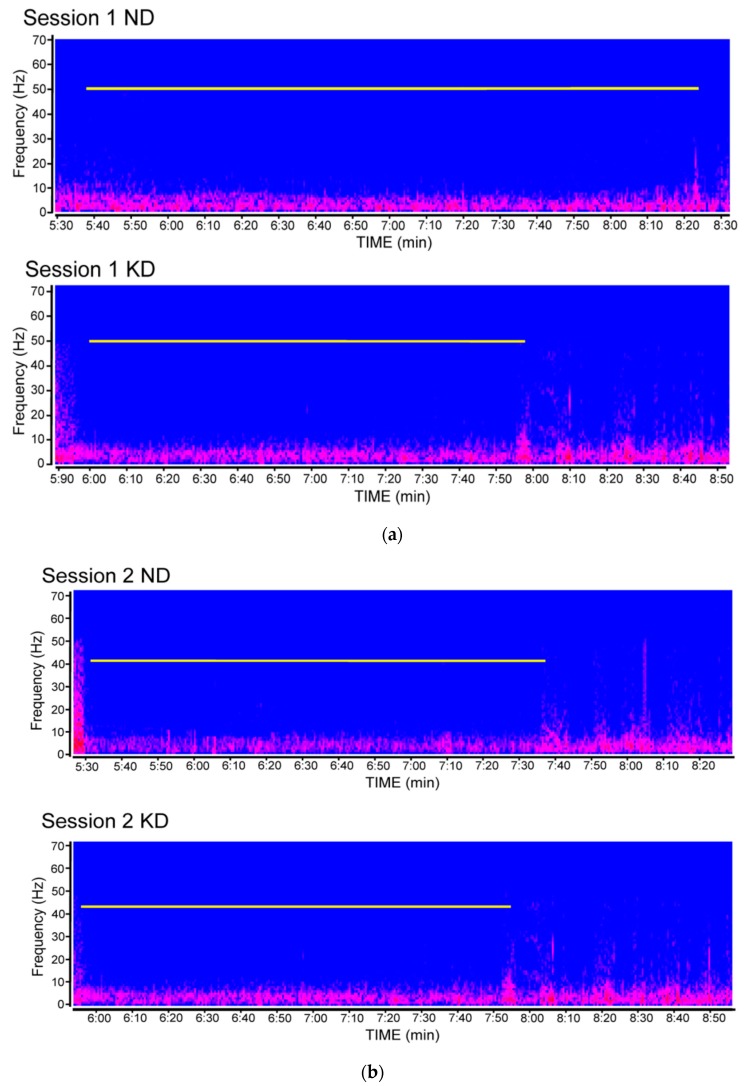
Compressed spectral array showing the duration of post-ictal depression in video electrocorticographic (ECoG) recordings obtained from mice fed the ketogenic diet (KD) or a normal diet (ND) and repeatedly tested in the 6-Hz corneal stimulation model. (**a**) ECoG traces illustrating the response detected in the first session of seizure induction. The white line indicates the duration of post-ictal depression. In (**b**), ECoG traces from mice stimulated in the second session of seizure induction. Spectral arrays were from different mice of each respective experimental group. Note that the difference observed in the first session is no longer detectable in the second session of 6-Hz corneal stimulation.

By analyzing the electrocorticographic (ECoG) traces (Figure 2a), apparently similar epileptiform events were recorded in the first session. As previously described [25], an ictal component followed by post-ictal flattening of the activity was found in both groups (Figure 3). Ictal activity lasted for a similar time interval in the two groups (Figure 2b), whereas the post-ictal depression was initially shorter in the KD group (*p* < 0.01, Holm–Šídák test; Figure 2c and Figure 3). Confirming our previous results [25], in the second session the period of post-ictal flattening was shortened in control mice maintained on the normal diet (ND) (*p* < 0.01 vs. session 1 of control group; Figure 2c), whereas no changes were present in mice maintained on the KD. Surprisingly, in the ECoG of mice fed the KD (Figure 2a) the ictal component of the electrographic seizure appeared to be more persistent in the second session compared to the first session (*p* < 0.01) (Figure 2b). This change was significant also when compared to values measured in control mice during the second session (*p* < 0.01; Figure 2b). Interestingly, a longer ictal activity was observed in the KD group in the further sessions of seizure induction, being significantly longer in the fourth compared to the first session of seizure induction in the same animals (*p* < 0.05; Figure 2b).

### 2.2. Seizures Worsened in Three Patients Maintained on the KD

The mean age of patients examined in our study was 7.0 ± 0.7 years. Female patients were 71% (Table 1).

**Figure 4:**
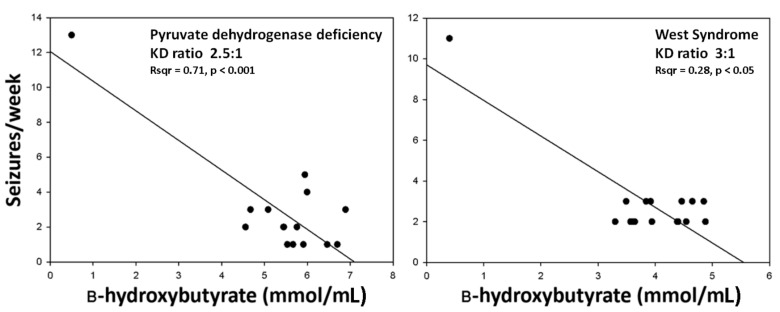
The relationship between ketosis and seizure reduction was analyzed by linear regression in two patients, whose characteristics are reported in Table 1 (#2 and #6).

The majority of patients (18/21, 86%) continued the KD for at least 3 months. The KD was started during hospitalization as a 1:1 ratio and then was progressively increased until an effective level of ketosis was achieved. All patients developed a ketosis ranging from 2 to 5 mmol/mL during the first days. Seizures were recorded by parents starting one month in advance and up to 15 weeks after the KD onset. By excluding patients who stopped the nutritional treatment (cases labeled as #10, #18, #21 in Table 1), we observed a greater than 50% decrease in the seizure frequency in 15 out of 18 patients (83%) who continued the KD for 3 months, hereafter indicated as responders. In these cases, a significant relationship was found between ketosis and seizure reduction, as exemplified in Figure 4. However, in 3 other patients (17%), the decrease in seizure frequency was less than 50%. Finally, in 3 patients (14% of all subjects) seizures worsened under treatment with the KD, but the worsening was transient in one of these cases.

**Figure 5:**
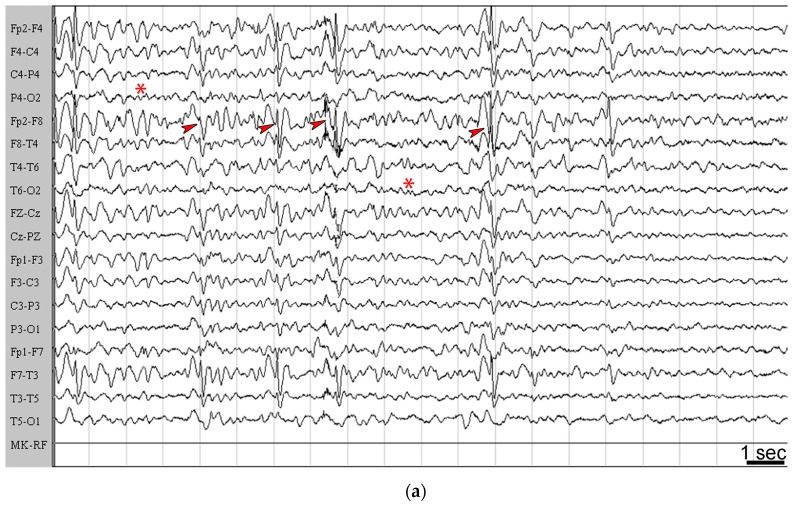

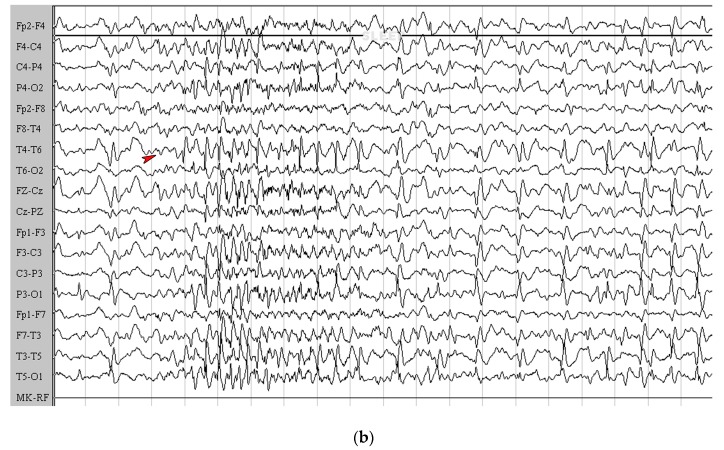
The sleep electroencephalogram (EEG) of patient #3 is illustrated. (**a**) EEG recorded before starting the ketogenic diet (KD). Note that a lack of spike and wave activities in the centroposterior areas is indicated by asterisks. Slow and sharp waves are frequently recorded from frontal regions (arrowheads), with a tendency to generalize and synchronize in bursts. (**b**) EEG obtained under treatment with the KD. Note that subcontinuous high-voltage sharp-waves are present (arrowhead), and that short ictal activity, which corresponded to minimal clinical expression (staring with slow rhythmic eye movements), is also detectable. EEG recordings were made by a 12 channel system (Galileo NT Suite Software). Images are presented on longitudinal reading montages. Amplitude, 20 µV/mm.

Specifically, in one patient, affected by Lennox–Gastaut syndrome associated with tuberous sclerosis complex, seizures presented with increased severity with no change in the frequency two weeks from the onset of the nutritional treatment. Then she recovered the therapeutic effect after an additional two weeks in which no change in the level of ketosis was observed. For this reason, this patient was nevertheless considered in the long-term as a responder (#3 in Table 1). The aggravation observed in this case is illustrated by the EEG recordings obtained before the onset of nutritional treatment (Figure 5a) compared to the EEG recorded after three weeks of KD treatment (Figure 5b), where it is possible to observe frequent high-voltage spike-waves, especially in the frontal right region, without a continuous or diffuse pattern and with spike waves that tend to assemble in brief electrical discharges or in electroclinical crisis. There was no clear explanation for this phenomenon.

**Figure 6:**
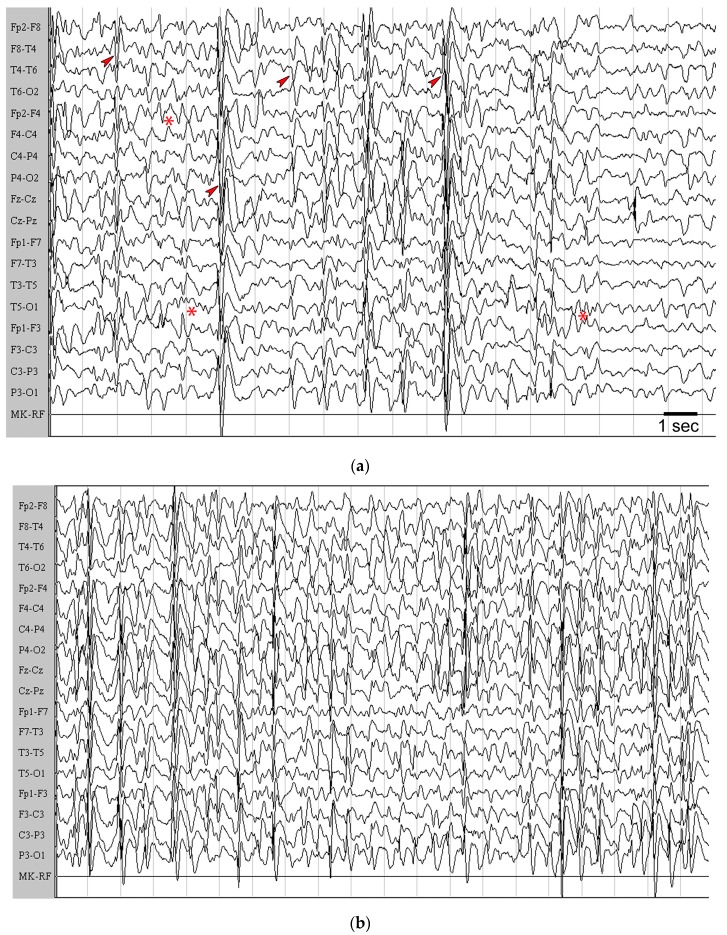
The awake electroencephalogram (EEG) of patient #18 is illustrated. (**a**) EEG before starting the ketogenic diet (KD). Note that a short sequence of posterior 5–6 Hz activity is detectable (asterisks). Then, this rhythm was interrupted by frequent high-voltage, generalized, and multifocal sharp-waves that tended to synchronize in short bursts (arrowheads), followed by a moderate reduction of the electrical amplitude. (**b**) Under treatment with the KD, the presence of continuous, high-amplitude, generalized sharp-waves and slow-waves starting from the frontal region is appreciable. These waves synchronized in short generalized bursts followed by mild suppression of basal activity. Note also that the physiological posterior rhythm was not present. EEG recording was made by a 12 channel system (Galileo NT Suite Software). Images are presented on longitudinal reading montages. Amplitude, 20 µV/mm.

In the other two cases, presenting with type I lissencephaly positive to LIS1 mutation, the aggravation was observed immediately after the KD administration. In these two children we noticed an increase in seizure frequency (≥25%) already during the first days of treatment (cases #10 and #18, Table 1). As shown in Figure 6 (only #18 is shown), a clear aggravation of interictal epileptic activity was detectable by the EEG, where diffuse and high voltage spike-waves appear to be more frequent without recovering baseline activity. In these cases, seizures were subcontinuously tonic, accompanied by cyanosis, and alternated with clonic shakes. All these changes disappeared when the KD was discontinued (not shown).

## 3. Discussion

In the present investigation, we describe various unreported changes occurring in mice that are fed with the KD and exposed to repeatedly induced seizures. We also identified three cases of paradoxically varied epileptic activity in patients affected by drug-resistant seizures and treated with the KD, in whom seizure frequency and/or severity were increased. To our knowledge, these clinical findings are also currently unreported. Overall, the major findings of our investigation are: (i) the prolonged electrographic epileptiform activity found in mice which are fed the KD and which experience repeated seizures; (ii) the concomitant failure to maintain the therapeutic effects of KD in the 6-Hz corneal stimulation model; (iii) the lack of any modulatory effect of the KD on epileptogenesis in our model; iv) the remarkable changes found in the EEG of patients experiencing a progressively severe clinical condition.

The 6-Hz corneal stimulation model has been suggested to be useful in evaluating the refractoriness to AEDs [19,28]. This latter is a required condition to propose the KD to patients affected by epilepsy. Furthermore, the 6-Hz corneal stimulation model was shown to reproduce the anticonvulsant properties of the KD by demonstrating that the induced ketosis was associated with an increase in the seizure threshold, or alternatively by showing that key components of the diet could reproduce the same effects [16,17,29,30,31]. We challenged the already demonstrated properties of the KD in our modified 6-Hz corneal stimulation model in which, instead of the threshold, we considered the severity and duration of the seizure. Surprisingly, we initially observed a beneficial effect of the KD at the first session of seizure induction. This finding was at odds with previous reports describing a lack of effects, or even worsening in the response to suprathreshold proconvulsive stimuli in animals which are fed the KD [14,15]. In all likelihood, differently investigated animal models could explain these discrepancies.

Rodents exposed to 6-Hz corneal stimulation were never reported to develop spontaneous seizures, even when daily kindled for weeks by using a more intense stimulation [19] or a higher frequency [32]. Consistently, our mice did not present seizures in the interstimulation period or after the four sessions of seizure induction. Thus, our protocol did not induce epilepsy, i.e., a condition in which unsolicited recurrent seizures can be observed. In particular, as the induced seizures were worse and worse by repeating the 6-Hz corneal stimulation, our model reproduced some features typical of the kindling procedure [33], but in presence of a fully effective stimulation. According to the definition of “secondary epileptogenesis” given to the progression in seizure severity observed in patients experiencing recurrent seizures [34], we hypothesized that our model could be useful in addressing the characteristics of seizures refractory to therapeutic treatments. Indeed, we were surprised to observe that the beneficial effects of the KD subsided at the second seizure induction and that, paradoxically, the electrographic discharge resulted in approximately double the duration of values found in control animals. This change accompanied the increased seizure severity observed in the KD group in the second compared to the first session of 6-Hz corneal stimulation, but no difference between the two investigated groups was apparent in the duration of both the motor and non-motor components of the seizure induced by 6-Hz corneal stimulation in the second session [25,28]. Thus, the prolongation of the electrographic discharge could be a specific feature of seizures resistant to KD treatment.

We characterized the repeated 6-Hz corneal stimulation model as a paradigm in which some aspects of epileptogenesis were reproduced. We previously found that a more severe seizure phenotype appears by repeating the seizure induction, and that this change is associated with the spreading of the electrographic epileptiform activity to the hippocampal CA1 region [25]. Although we did not take readings from the hippocampus in the present experiments, we observed that the KD was not able to prevent the appearance of more severe seizures in mice exposed to repeated 6-Hz corneal stimulation. So, the KD failed in exerting an antiepileptogenic effect in our model. This interpretation is supported by previous results obtained in mice treated with the anticonvulsant peptide EP-80317, which was instead able to delay the appearance of more severe seizures up to the third session of corneal stimulation [26]. Of course, the negative findings obtained in the 6-Hz model do not exclude the possibility that other antiepileptogenic effects could be revealed in different models. Indeed, the KD was shown to be effective by using both electrical and chemical kindling procedures, as well as in the pilocarpine model [21,22]. On the other hand, lack of efficacy or partially beneficial effects were also reported for the KD, respectively, in the lithium-pilocarpine model [24] or in genetically epileptic mice [23], thus suggesting a strong influence of the investigated animal model on the efficacy of KD administration on epileptogenesis.

In three patients, we observed a paradoxical effect of the KD. This phenomenon resembles that described for AEDs [10]. Since we carefully monitored the ketosis induced by the KD, the observed worsening was unexpected. Blood levels of AEDs were also carefully monitored in our patients, and no additional complications were recorded. Thus, there was no clear explanation for the paradoxical response observed in the three patients treated with the KD. However, in one of them affected by the Lennox–Gastaut syndrome the aggravation was only transient and did not prevent the continuation of the treatment. On the contrary, the KD had to be stopped in the two patients affected by lissencephaly. We considered the possibility that the presence of this specific disease could be a reason for the worsening. However, patients affected by the same condition were successfully treated with the KD by other investigators [35]. The analogue paradoxical responses reported for AEDs were also without a clear explanation, as in the case of our patients, and they were generally interpreted as an adverse reaction to the mechanism of the prescribed AED, occurring only in some patients [10]. Indeed, further investigations are required to understand the reasons why the KD might cause a paradoxical worsening. To this aim, the 6-Hz model of repeated corneal stimulation may provide an experimental tool to give these answers.

## 4. Materials and Methods

**Figure 7:**
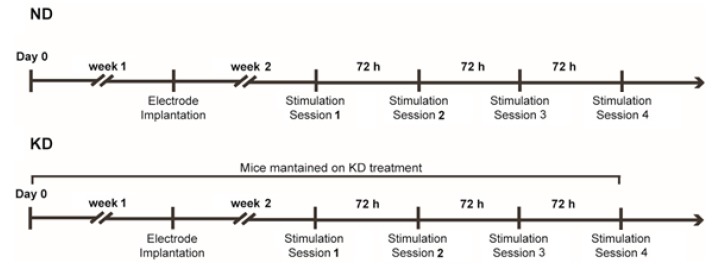
Timeline of the experiment with mice maintained on the normal diet (ND) or on the ketogenic diet (KD) and exposed to various sessions of 6-Hz corneal stimulation.

A total of 18 (four week-old) male CD-1 mice (Charles River, Calco, Italy) were used in this study. They were housed in a specific pathogen-free facility under a controlled environment with *ad libitum* access to water and food. Mice were randomly assigned to two distinct treatment groups. Of these mice, nine were fed the ND. Nine mice, instead, received the KD for two weeks (Teklad TD.96355, Harlan Italy) before being subjected to the 6-Hz corneal stimulation test (Figure 7). The KD was also administered during the testing period and consisted of (g%, *w*/*w*) 67.43 fats, 15.08 proteins, and 0.54 carbohydrates, resulting in a fats-to-proteins+carbohydrates ratio of 4.3:1. The caloric density of the KD was 6.69 kcal g^−1^ (fats: 90.66%; proteins: 9.01%; carbohydrates: 0.32%). Mice were weighed once a week to monitor body growth.

For electrode implantation, mice were anesthetized with ketamine + xylazine (150 + 10 µg/g). Guiding holes were drilled and epidural electrodes (stainless steel Ø = 1 mm; PlasticsOne, Roanoke, VA, USA) were implanted in the frontal (bregma 0 mm, 3 mm lateral from midline) and occipital cortices (bregma −3.5 mm, 3 mm lateral from midline) of the right hemisphere [25,26,27]. One electrode was implanted below lambda on the midline and used as reference. At the end of the surgery, gel containing 2.5 g lidocaine chloride, 0.5 g neomycin sulfate and 0.025 g fluocinolone acetonide (Neuflan^®^ gel; Molteni Farmaceutici, Florence, FI, Italy) was applied to reduce pain and the risk of infection.

Mice were placed in cages without a cover to allow a cable to connect the headset and preamplifiers. Electrical brain activity was filtered (0.3 Hz high-pass, 500 Hz low-pass), acquired at 1 kHz per channel, and stored on a personal computer as the mathematical subtraction of traces of recording electrodes minus traces of reference electrode (only for epidural electrodes), using a PowerLab8/30 amplifier connected to four BioAmp preamplifiers (ADInstruments, Dunedin, New Zealand). Videos were digitally captured by a camera connected to the computer and synchronized to the ECoG traces by LabChart 7 Pro internal trigger.

Mice were stimulated once and let to recover for three days before being stimulated for a second time. Corneal stimulation was as previously described [25,26,27]. Briefly, ocular anesthetic (0.4% oxybuprocaine hydrochloride eye drops, Novesin, Laboratoires Théa, 63000 Clermont-Ferrand, France) was applied 10 min before stimulation. Stimulation (fixed current intensity of 32 mA, pulse width of 0.2 msec, duration of 3 sec, frequency of 6 Hz) was delivered via external non-invasive corneal electrodes connected to a stimulator (ECT Unit 5780; Ugo Basile, Comerio, Italy), as shown by Castel-Branco et al. [36].

All experiments were in compliance with the European Directive 2010/63/EU and carried out according to the national guidelines on animal experimental research of the Italian Ministry of Health (DM 92/2013). The University of Modena and Reggio Emilia Ethics Committee approved the study protocol. All efforts were made to refine procedures to improve the welfare and to reduce the number of animals that were used.

ECoG traces were offline digitally filtered (band-pass: high 50 Hz, low 1 Hz) and manually analyzed using LabChart 7 Pro software v7.3.8 (ADInstruments) by blind to treatment expert raters. Seizures were characterized by epileptiform ECoG patterns occurring right after the stimulus artifact. ECoG patterns characterized by trains of 150–250 ms long spikes with amplitudes at least twice as the previous 2 sec baseline were considered as electrographic seizures.

Patients with drug-resistant seizures admitted to the pediatric neurology service between April 2011 and February 2017 and treated with the KD were retrospectively examined. These patients were treated with the KD due to (i) a failure of drug treatment with two or more appropriately chosen AEDs; (ii) good compliance with AED administration; (iii) lack of alterations of serum amino acids, urine organic acids, acylcarnitine, lactate, pyruvate, and ammonia blood levels. They received the nutritional treatment by starting with a 2:1 ratio of lipids and proteins + carbohydrates and, after few days, shifting to a 3.5/4:1 ratio [37]. Routine laboratory and encephalographic investigations were fully available for at least 1–3 months of treatment. Blood levels of AEDs were also recorded. The final study was conducted on a cohort of 21 subjects with ages ranging from 3 to 15 years. Data about demographic features, clinical characteristics, diagnostic findings, therapeutic interventions, and clinical outcomes are reported in Table 1.

The ketone body β-hydroxybutyric acid was measured in fasting blood samples obtained by isoflurane anesthetized mice, one day after the testing procedure. Precisely, trunk blood was collected after decapitation of deeply anesthetized mice. One drop of blood was adsorbed on a test strip and read with the Precision Xtra Blood Ketone Meter (Abbott Diabetes Care, Rome, Italy). In patients, fasting glucose and ketone basal values were noted during hospitalization; after that, parents reported glycaemia and β-hydroxybutyric acid levels three times/day/week up to 15 weeks.

β-hydroxybutyric acid levels were compared by Student’s *t* test. Linear regression analysis was applied to investigate the relationship between ketosis and seizure reduction. Fisher’s exact test was used to compare mice that developed seizures with loss of posture with those that maintained posture control, in the two different groups of treatment. Seizure duration and electrographic recordings were compared using two-way analysis of variance, considering the different diet as the between factor and the session of 6-Hz corneal stimulation as the within factor. The Holm–Šídák test was used for multiple comparisons. All statistical analyses were carried out using Sigmaplot 11 (Systat Software, San Jose, CA, USA). Data are presented as mean ± standard error of the mean (SEM) and were regarded significantly different at *p* < 0.05.

## Figures and Tables

**Table 1:** Demographic and clinical features of patients treated with the ketogenic diet (KD). Abbreviations: AEDs, antiepileptic drugs; CBZ, carbamazepine; CLB, clobazam; CZP, clonazepam; ESM, ethosuximide; GVG, vigabatrin; LEV, levetiracetam; LTG, lamotrigine; NZP, nitrazepam; PB, phenobarbital; PCDC, pyruvate dehydrogenase complex deficiency; RFN, rufinamide; TPM, topiramate; VPA, valproate; ZNS, zonisamide.

Code	Gender	Age	Diagnosis	Type of Seizure	AEDs	Type of KD	Response (Reduction in Seizure Frequency)	Transient Side Effects
#1	F	5	Respiratory chain defect I-III complex	Spasm	CBZ, NZP, LEV	Classic 2:1	88%	Hypercholesterolemia, hyperoxaluria
#2	F	4	Suspected PDCD	Tonic-clonic, spasm	NZP, LEV	Classic 3:1	92%	Nausea, vomiting
#3	F	9.6	Lennox–Gastaut syndrome	Spasm, drop attack, tonic-clonic	LEV, RFN, NZP	Classic 4:1	Transient worsening, then 78% reduction	Hypertriglyceridemia, acidosis
#4	F	5	Congenital hydrocephalus	Drop attack, myoclonus, absence	TPM, LEV	Classic 2:1	94%	Hypercholesterolemia
#5	M	5.9	Suspect of GLUT1 deficiency	Tonic-clonic, spasm	TPM, CZP	Classic 4:1	52%	Hyperphosphatemia
#6	M	5.9	Epileptic encephalopathy	Tonic-clonic, spasm	GVG, VPA, TPM	MCT 3:1	97%	No
#7	F	5.4	Partial trisomy for chromosome 13	Tonic-clonic, drop attack	VPA, NZP, RFN	MCT 3.5:1	96%	Nausea, vomiting
#8	M	5.3	Epileptic encephalopathy	Tonic-clonic	TPM, ESM	Classic 3:1	95%	No
#9	M	4.7	Suspected GLUT1 deficiency	Myoclonus, tonic-clonic	GVG, CBZ, NZP	Classic 3:1	97%	Hypercholesterolemia
#10	M	4	Lissencephaly type 1	Tonic-clonic, spasm	CZP, PB, CBZ	Classic 4:1 + MCT	Worsened	Nausea, vomiting
#11	F	3.9	Dravet syndrome	Myoclonus	TPM, CZP, CLB	Classic 2:1	48%	No
#12	F	9.3	GLUT1 deficiency	Absence, spasm	VPA	Classic 2.5:1	100%	Hypercholesterolemia
#13	M	3	Epileptic encephalopathy	Tonic-clonic, drop attack	TPM, CZP, CLB	Classic 4:1	45%	No
#14	F	9.6	Epileptic encephalopathy	Absence, spasm	VPA, LTG, CLB	Classic 4:1	91%	Constipation
#15	M	8.2	Suspected PDCD	Tremor	NZP, LEV	Classic 3:1	53%	No
#16	F	6	Cerebral cortex malformation	Absence, tonic-clonic	LEV, LTG	MCT 3:1	63%	Constipation
#17	F	6.5	West Syndrome	Tonic-clonic	TAS, CBZ, ESM	Classic 2:1	0%	No
#18	F	7.1	Lissencephaly type 1	Tonic-clonic, spasm, absence	VPA, CLB, RFN	Classic 2:1	Worsened	No
#19	F	14	Cryptogenic generalized epilepsy	Tonic-clonic, absence	LEV, CZP, PB	Classic 3:1	73%	No
#20	F	14.3	Epileptic encephalopathy	Tonic-clonic	LEV, ZSN, NZP	Classic 4:1	63%	Hypercholesterolemia, weight gain
#21	F	10	Epileptic encephalopathy	Tonic-clonic, absence	LTG, LEV	MCT 3:1	51%	Vomiting, constipation
